# Preoperative Quality of Life as Predictor of Postoperative Early Recurrence and Overall Survival in Patients with Pancreatic and Periampullary Adenocarcinoma

**DOI:** 10.1007/s12029-026-01473-3

**Published:** 2026-05-21

**Authors:** Carsten Palnaes Hansen, Lise Munk Plum, Jan Henrik Storkholm, Hans-Christian Pommergaard

**Affiliations:** 1 Department of Digestive Diseases, Transplantation and General Surgery, Rigshospitalet, Copenhagen University Hospital, Copenhagen, Denmark; 2 Hepatic Malignancy Surgical Research Unit (HEPSURU), Copenhagen University Hospital, Rigshospitalet, Copenhagen, Denmark; 3 Section for Enhanced Recovery after Surgery and Disease (ERASD), Rigshospitalet, Copenhagen University Hospital, Copenhagen, Denmark; 4 Department for Clinical Medicine, University of Copenhagen, Copenhagen, Denmark

**Keywords:** EORTC, Quality of life, Postoperative complications, Pancreatic cancer, Survival

## Abstract

**Background:**

The purpose of the study was to investigate if preoperative assessment of quality of life (QoL) in patients with pancreatic and periampullary cancer can identify patients with reduced recurrence free and overall survival after pancreatic surgery.

**Methods:**

A prospective study of patients planned for pancreatic surgery due to pancreatic and periampullary tumors. Participants completed the European Organization for Research and Treatment of Cancer (EORTC) C30 and PAN26 questionnaires preoperatively.

**Results:**

Of 112 participants, 95 patients had pancreatic or periampullary adenocarcinoma, and 17 patients had benign pathology. The operations included pancreaticoduodenectomy (79), left pancreatectomy (1), total pancreatectomy (16), and exploratory laparotomy (16) due to locally advanced and/or metastatic disease. Patients with adenocarcinomas had a lower EORTC score in functional and social domains than patients with benign disease and a higher score within physical symptoms in both questionnaires. This was most pronounced in patients with advanced diseases. Patients with advanced disease or metastases and patients with recurrence within 6 months had significant deviations in several domains compared to patients without recurrence. However, there was only a weak relation between preoperative QoL scores and disease specific survival (DSS) and recurrence free survival (RFS) and only night pain remained in the model after multivariate regression (*p* = 0.013). Adjusted analyses for co-variates only showed association between outcome and lymph node stage. The overall survival, DSS and RFS was 30.9, 40.5, and 15.0 months, respectively. The recurrence rate in patients with PDAC and periampullary adenocarcinoma was 67% during the observation time, with a median time to recurrence of 9.0 (range 3.0–67.0) months.

**Conclusion:**

Among patients undergoing surgery for pancreatic and periampullary tumors, preoperative quality of life according to EORTC scores differed between patients with malignant and benign disease but was only weakly associated with survival outcomes.

## Introduction

Pancreatic ductal adenocarcinoma (PDAC) is among the four leading causes of cancer related death and may become the second most common cause by 2030 [1, 2]. Although 5-year survival rates have improved, overall survival after surgical resection remains about 20%, varying by stage and oncologic therapy. Less than 25% of patients are eligible for surgery at diagnosis; those with advanced or metastatic disease have 5-year survival rates of only 9% and 2%, respectively.

Postoperative mortality is less than 2% in high-volume centers, so apart from medical conditions the long-term mortality after surgical resection is primarily due to recurrent disease [3, 4]. More than 80% of operated patients will develop distant metastases, thus pancreatic cancer is a systemic disease even in patients with localized tumors.

A thorough preoperative assessment of patients before surgery for PDAC is important to ensure a satisfactory course after surgery. Apart from imaging diagnostics, biochemical screening and physical examination, patient reported outcome (PRO) questionnaires may be used to give an impression of patients’ preoperative physical and psychological condition and provide health care professionals with additional data on operability and prognosis.

Quality of life (QoL) questionnaires used in patients with cancer have become central in the assessment of the health outcomes of therapeutic programs, and from the beginning were neither meant as diagnostic nor prognostic tools. However, questionnaires may provide additional data in the evaluation of patients’ prognosis as supplemental to performance score (PS) [5]. QoL evaluation may, therefore, help the clinician to find patients with early recurrence and arrange further diagnostic examination. The value of the questionnaires in this setting has been studied, but with different outcomes [6]. During the first months after pancreatic surgery, most patients will have a recovery period with a significantly reduced QoL [7–9], but some may have persistently reduced QoL masking disseminated disease.

Most studies of QoL have been performed postoperatively or as comparative studies between preoperative and postoperative outcomes while there are only a few studies that have used preoperative data from QoL questionnaires prognostically in patients with PDAC [10, 11]. Recurrence within the first 12 months is observed in more than 50% of patients who have undergone surgery for PDAC [12]. Preoperative assessment of resectable PDAC for predictors of disseminated disease may guide the selection of patients suitable for upfront resection or those that may benefit from neo-adjuvant chemotherapy.

The primary aim of this study was to investigate if patients’ QoL based on standardized questionnaires before surgery for presumed or diagnosed PDAC or periampullary adenocarcinoma could help identify patients with disseminated disease undetectable on pre-operative imaging resulting in early postoperative recurrence. A secondary aim was a long-time follow-up with causes of death in all patients who underwent pancreatic surgery.

## Methods

### Patients

Patients undergoing pancreatic surgery for suspected malignant or premalignant disease were included in this prospective study. The period of recruitment was 1. May 2015 to 31. December 2016, and patients were followed until 30. April 2025 or death.

Patients were referred from local hospitals after diagnostic imaging, which as a minimum included a triple-phase multidetector-row computed tomography and if needed supplementary magnetic resonance imaging and/or endoscopic ultrasonography. Computed tomography scans were performed according to a standardized protocol to ensure uniform quality, and the images were electronically forwarded and evaluated by radiologists specialized in hepato-pancreato-biliary (HPB) imaging and discussed at a multidisciplinary tumor board meeting (MDT). Given the seriousness of PDAC there is a need for timely treatment. Therefore, eligible patients were booked for surgery prior to consultation. Before the operation, a pancreatic surgeon and an anesthetist evaluated the patient’s operability in the out-patient clinic, and a dedicated counselling providing the patient with information about operation and goals for recovery was given.

Physical status and comorbidity were classified according to the American Society of Anesthesiologists (ASA) and the Royal College of Surgeons Charlson score.

### Surgical Procedures

All surgical procedures were standardized and included pancreatico-duodenectomy (Whipple’s operation: resection of the pancreatic head, duodenum, antral part of the stomach, gall bladder, and the common bile duct), total pancreatectomy as Whipple’s procedure including splenectomy, and distal pancreatectomy with resection of the body and tail of the gland including splenectomy.

### Postoperative Complications

Relevant postoperative surgical complications include leakage from the pancreatic, bile or gastrojejunal anastomosis, intraabdominal hemorrhage and abscess formation, gastrointestinal bleeding, or other complications with severe or fatal outcomes.

All complications were scored according to the Clavien–Dindo classification for surgical complications and the definitions set by the International Study Group of Pancreatic Surgery (ISGPS) [13, 14].

The definition of postoperative pancreatic leak is a concentration of amylase three times higher in drain fluid than serum amylase on, or after the third postoperative day. Elevated drain amylase alone (Grade A) has no clinical impact and was not recorded in this study. Grade B involves a change in clinical management, and in Grade C, intensive care and/or surgical intervention is needed. Bile leak was defined as pancreatic leak except for bile instead of amylase in drain fluid.

Post-pancreatectomy hemorrhage Grade A has no clinical impact and is not recorded in this study. In Grade B hemorrhage therapeutic actions such as transfusion, relaparotomy or embolization will be needed. Grade C is potentially life threatening and leads to immediate intervention.

### QoL questionnaires

QoL was defined as the patient’s perceived physical, mental, and social health-status measured by the European Organization for Research and Treatment of Cancer (EORTC) QLQ-C30 and QLQ-PAN26 questionnaires. The QLQ-30 is a cancer health-related QoL questionnaire designed to measure physical, psychological, and social functions in cancer patients [15]. It consists of 30 questions and covers five functional scales (physical, role, emotional, cognitive, social, and global health) and three symptom scales (fatigue, nausea and vomiting, and pain) all domains with a number of items, and finally five single items (sleep disturbance, anorexia, constipation, diarrhea, and financial problems).

The QLQ-PAN26 was developed to measure health status and disease burden especially among pancreatic cancer patients [16]. The questionnaire contains 26 items related to PDAC symptoms, treatment side effects, and emotional issues.

The QLQ-C30 and QLQ-PAN26 raw scores were calculated using the EORTC manuals [17]. Estimation of the average of the items that contribute to the scale gives the raw score in percentage, which ranges from 0 to 100. A high score represents a higher or better level of functioning, or a higher or worse level of symptoms. We defined cut-off values between low and high score to be </≥ 66.66%.

After thorough instruction by a trained interviewer, the patient received the questionnaires at the first pre-operative outpatient visit and submitted the completed form before surgery took place.

Only patients who had pancreatic resection for presumed or diagnosed PDAC or periampullary adenocarcinoma were included. Patients who had neoadjuvant oncologic therapy were excluded. Patients were divided postoperatively into four sub-groups: resected benign tumor, resected cancer with and without recurrence, exploratory surgery due to locally advanced disease or dissemination.

### Data Collection

Apart from the questionnaires, data were collected from our prospectively maintained clinical database of pancreatic operations, from the electronic hospital medical record systems Orbit and EPIC, the Danish National Pathology Data Registry, and from the National Register of Death. All Danish Nationals have a unique central person registration number that enables searching of health data.

The following baseline characteristics were collected: age, sex, Charlson comorbidity score without correction for age, ASA score, surgical procedure, histopathology, tumor size, stage, resection margin or surface (R0 > 1 mm, R1 ≤ 1 mm), length of hospital stay (LOS) from operation to discharge, and adjuvant chemotherapy.

Outcomes were recorded as postoperative complications, LOS, 30- and 90-days assessed mortality, OS, recurrence free survival (RFS), and disease specific survival (DSS). OS is defined as time from surgery to death from any cause, RFS is length of time from surgical treatment until diagnosis of recurrence, and DSS time from surgery until death from PDAC and periampullary adenocarcinoma excluding deaths from other causes.

After operation for adenocarcinoma, all patients were followed at regular intervals with show ups or via telephone during the two years. Patients were monitored by their clinical condition, CA 19 − 9, and CT scans if recurrence was suspected. Follow-up ended when the patient died or the study was terminated. Estimation of remaining life expectancy was performed based on Statistics Denmark’s tables for life expectancy 2015–2016 and corrected for region of residence (www.dst.dk).

### Statistical Analysis

QoL data are presented as mean and 95% CI of mean, survival data median and range. Categorical data are presented as numbers or percentages and were analyzed with Fisher’s exact test and the Mann-Whitney test. The Kaplan–Meier method was used to estimate OS, DSS and RFS. QoL summary scores in relation to recurrence were analyzed by logistic regression and results evaluated by the likelihood ratio test and the area (AUC) under the receiver operating characteristic curve (ROC). Cutoff points of relevant scores were determined by Youden statistics and index of union (IU) [18, 19]. Standardized effect size for measuring the difference between two group means was made with Cohen’s test [20]. QoL scores in relation to DSS and RFS were analyzed with univariate Cox proportional hazard regression. *p*-values < 0.25 in the univariate analysis were used in the multivariate Cox model by stepwise regression with backward elimination [21], and the Akaike information criterion (AIC) was used to compare the qualities of each statistical model to each other. A *p* < 0.05 was considered significant. Statistical analysis was performed with GraphPad Prism version 6.05 (GraphPad^®^, La Jolla, CA). The study was reported according to the STROCSS guidelines [22].

### Ethics

Written informed consent from patients were obtained. Only patients fluent in spoken and written Danish were included. The project was notified on September 30, 2015, in accordance with the umbrella notification scheme between the Capital Region of Denmark and the Danish Data Protection Agency. The Danish Data Protection Agency approved the data management (2012-58-0004 and RH-2015-136, med I-Suite nr: 04017). According to Danish legislation, quality assurance research is exempted from ethical approval. The study was performed in compliance with the Helsinki Declaration.

## Results

**Fig. 1:**
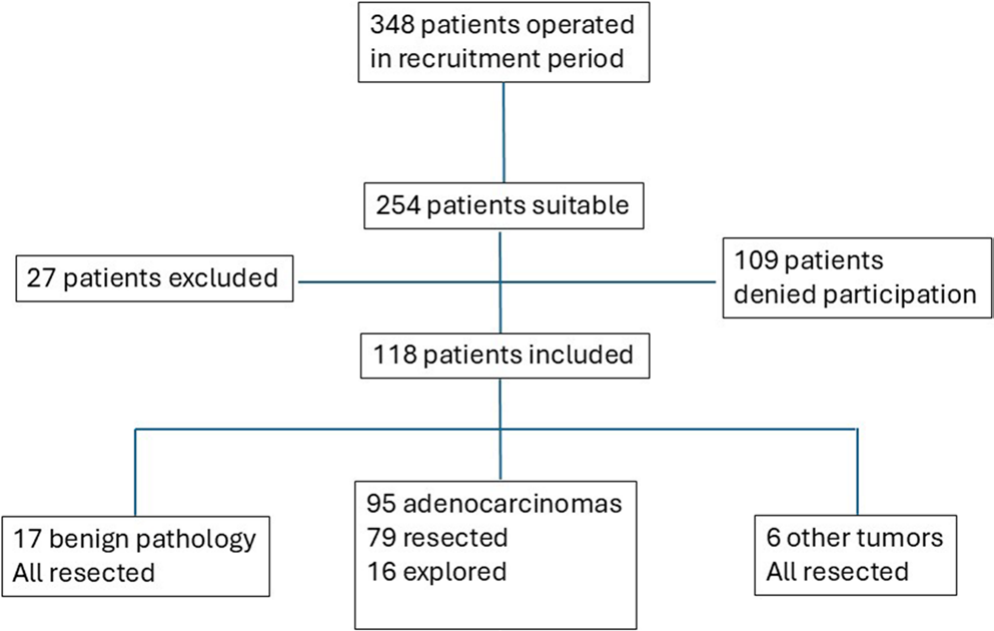
Flow-chart of participants

During the recruitment period, 348 patients were operated on for pancreatic and periampullary tumors. Of the 254 patients initially found suitable to participate in the project, 142 patients were excluded (109 patients denied participation, 27 patients were excluded for other reasons, and six patients had other malignant diseases and were excluded after the operation) (Fig. 1). Sociodemographic and clinical characteristics of the 112 remaining participants who completed the questionnaire are given in Table 1.

**Table 1:** Sociodemographic and clinical characteristics of 96 patients with carcinoma and benign pathology, who underwent pancreatic resection

Age, years (mean, range)	66 (29 – 82)
Gender (M/F)	57/39
Employment^1^	
Employed	34 (36%)
Un-employed/retired	60 (44%)
Educational level^2^	
Elementary	26 (31%)
Higher	51 (61%)
University	7 (8%)
BMI (mean)	22.2
BMI < 18.5	15.0 (22%)
ASA score	
1	23 (24%)
2	51 (53%)
3	21 (22%)
4	1 (<1%)
Charlson score	
0-1	53 (55%)
2-3	37 (39%)
> 3	6 (6%)
Comorbidity	
Heart	29 (30%)
Diabetes	12 (13%)
Lungs	5 (5%)
Active smoker	26 (27%)
Alcohol/week (≥ 7 units^2^A)	19 (20%)
Symptoms	
Jaundice	45 (47%)
Pain	43 (45%)

^1^Reported

^2^1 unit is 12 gram of alcohol

Ninety-six patients underwent pancreatic resection, and 16 patients had exploratory surgery due to unresectable adenocarcinoma (Table 2). Pathological examination after the operation revealed 95 patients with PDAC or periampullary carcinoma, and 17 patients with benign pathology. Patients with periampullary adenocarcinoma with pancreatico-biliary differentiation were regarded as PDAC while adenocarcinoma with intestinal morphology were regarded as duodenal cancer.

**Table 2:** Surgical procedure and tumor pathology of 96 patients who had pancreatic resection due to carcinoma or benign pathology

Procedure	
Pancreaticoduodenectomy	79
Total pancreatectomy	16
Distal pancreatectomy	1
Pathology	
Adenocarcinoma	79
Pancreas	63
Periampullary	16
T1-T2	12
T3	78
T4	6
N0	23
N1	56
M0	77
M1	^1^2
Benign	17
IPMN	10
Pancreatitis	5
Pseudopapillary	1
Duodenal adenoma	1

^1^Para-aortic lymphnode 16

^2^A Corresponding 1

Nineteen patients developed 22 postoperative surgical complications (Table 3). Two patients had severe medical complications. These included one patient, who had a pacemaker because of postoperative A-V block, and another patient who died at ICU from respiratory failure due to chronic obstructive lung disease. This was the only in-hospital death.

**Table 3:** Surgical complications after pancreatic resection due to adenocarcinoma and benign disease

	Adenocarcinoma	Benign		
N	79	17		
Hospital stay/days(mean, range)	10 (6-40)	11 (7-23)		
Complications			Management	Dindo-Clavien score
Pancreatic fistula	1	1	PTC guided drain and conservative treatment	3b
Bile leak	5		PTC guided drain and conservative treatment	3b
Gastro-enteroanastomosis leak	1		Conservative treatment	2
Gastrointestinal bleeding	1		Gastroscopic hemostasis	3b
Intraabdominal haemorrhage	1		Intervention radiology with coiling	3b
Intraabdominal abscess		1	Ultrasound guided drainage	3a
Wound complications	10	1	Debridement and re-suture	3b

*PTC* percutaneous transshepatic cholangiography

There was no significant age difference among the scores in the two questionnaires, and the only significant gender difference in the answers was within the domain “sex life” in patient who had surgical resection for adenocarcinoma, where women scored a lover value (less impaired function) in the PAN26 than men, 35.90 (CI 18.90–52.89.90.89) vs. 58.33 (CI 46.54–70.13) (*p* = 0.031). However, we did not include sex life in the general evaluation.

When the QoL scores were divided into patients with benign disease (*n* 17) and adenocarcinomas (*n* 95), patients with adenocarcinomas had a lower QLQ-C30 score in domains like global, role (limitations in performing work or other daily activities), and social functions than patients with benign disease (Table 4). Moreover, patients with adenocarcinoma had a higher score within fatigue and digestive problems with related symptoms in both questionnaires. Pain was not a prominent symptom, and only nocturnal pain was significantly lower among patients without recurrence in the QLQ-PAN26 questionnaire (*p* = 0.015).

**Table 4:** Preoperative EORTC scores in patients who underwent operation

	No malignancy	Pancreatic and periampullary adenocarcinoma					
	Resection (N17)	Explorative surgery (N16)	Resection (N79)				
QLG-C30			No recurrence (N 26)	Recurrence (N 53)	(*p*)A	(*p*)B	(*p*)C
Global	75.6 (61.9-89.2)	49.0 (31.1-66.8)	50.6 (42.1-59.2)	58.2 (51.3-65.0)	0.012	0.003	
Functionally							
Physical	94.5 (90.1-98.9)	67.1 (52.7-81.4)	79.2 (71.2-87.3)	81.3 (75.2-87.3)	< 0.002	< 0.004	< 0.033
Role	88.2 (73.5-100)	50.9 (29.2-70.8)	52.6 (38.1-67.0)	59.4 (49.1-69.8)	< 0.001	< 0.001	< 0.001
Cognitive	90.2 (81.6-98.8)	69.8 (55.6-84.1)	84.0 (75.6-92.4)	79.5 (72.1-86.9)	0.013		
Social	87.3 (73.2-100)	56.5 (35.8-77.2)	82.7 (71.7-93.7)	76.7 (68.5-85.0)	0.009		
Emotional	71.6 (58.4-84.7)	67.7 (53.0-82.4)	68.6 (58.2-78.9)	76.3 (70.7-81.9)			
Symptoms							
Pain	16.7 (4.6-28.8)	40.6 (23.5-57.8)	21.8 (12.9-30.7)	23.2 (14.9-31.7)	0.011		
Fatigue	23.6 (13.5-33.7)	50.0 (31.3-68.7)	46.2 (32.5-59.8)	43.0 (34.4-50.6)	0.039	0.029	0.004
Loss appetite	13.7 (1.5-25.9)	52.1 (28.7-75.4)	35.9 (21.2-50.6)	39.0 (29.3-48.7)	0.005	0.034	0.005
Nausea	3.9 (0.1-8.7)	27.1 (10.0-44.2)	16.7 (7.0-26.4)	12.6 (7.3-17.9)	0.008	0.021	0.070
Diarrhea	7.8 (1.8-17.5)	18.6 (4.3-33.2)	32.1 (17.1-47.0)	17.9 (11.0-24.9)		0.017	
Constipation	5.9 (3.2-14.9)	16.7 (2.2-31.1)	9.0 (2.9-15.1)	18.2 (10.7-25.8)			
Dyspnoea	13.7 (5.0-22.4)	25.0 (6.1-43.9)	16.7 (4.5-28.9)	13.2 (6.9-19.5)			
Sleep disturbance	17.7 (7.0-28.3)	43.8 (25.7-61.8)	37.2 (23.3-51.1)	34.0 (24.3-43.6)			
PAN26							
Pancreatic pain	18.6 (8.8-28.5)	35.9 (24.1-47.8)	20.8 (12.7-28.3)	24.7 (19.0-30.4)	0.047		
^1^Back pain	19.6 (6.0-33.2)	33.3 (16.2-50.5)	25.6 (11.2-40.1)	27.0 (18.6-35.5)			
^1^Night pain	17.7 (5.4-29.9)	27.8 (13.8-40.4)	7.9 (0.2-16.1)	17.6 (10.7-24.5)			
Digestive function	15.7 (1.0-30.4)	58.3 (38.1-78.6)	35.3 (21.5-49.0)	34.9 (25.0-44.8)	< 0.001	0.016 =	0.026
Altered bowel habits	9.8 (1.2-18.4)	21.9 (11.8-32.0)	30.8 (16.8-44.7)	27.6 (19.2-36.0)	0.007	0.034	0.025
Flatulence	21.6 (9.5-33.6)	25.0 (12.9-37.1)	23.8 (10.1-37.5)	32.0 (22.1-41.9)			
Hepatic symptoms	8.8 (2.0-15.7)	18.7 (6.2-31.3)	32.7 (16.6-48.8)	41.1 (29.8-52.2)			0.006
Change of taste	11.8 (1.4-22.2)	45.8 (20.0-71.7)	23.1 (11.2-35.0)	24.7 (15.1-34.2)			
Dry mouth	7.8 (1.8-17.5)	52.1 (32.7-71.5)	28.6 (14.0-43.2)	34.0 (25.5-42.5)	< 0.001	0.005	0.006
Weakness	9.8 (0.3-19.9)	35.5 (15.5-55.4)	22.2 (11.1-33.3)	25.6 (18.2-33.1)	0.039	0.038	0.008
Low activity	17.7 (0.4-34.9)	50.0 (28.3-71.5)	38.1 (22.0-54.2)	39.6 (29.9-49.3)	0.027	0.036	0.011

p values are differences between patients with benign disease and patients who had (A) exploration, (B) no recurrence, and (C) recurrence of adenocarcinoma

^1^Subdivision of domain pancreatic pain

Between patients with unresectable vs. resectable disease regardless of recurrence, there was only a significant difference in scores corresponding to social function and pain in the QLQ-C30 (*p* = 0.025 and 0.020, respectively) and night pain in the QLQ-PAN26 questionnaire (*p* = 0.043).

Comparison between patients without recurrence and patients with advanced disease and early recurrence within 6 months showed significant differences in the QLQ-C30 domains cognitive, social function and pain, and in the QLQ-PAN26 domains pancreatic pain, night pain, and dry mouth (Table 5).

**Table 5:** Logistic regression of preoperative EORTC QLQ-C30 and QLC-PAN26 scores in patients with pancreatic and periampullary adenocarcinoma without recurrence (N 27) and patients with unresectable tumors or recurrence within six months after pancreatic resection (N 28)

	No recurrence	Advanced/Early recurrence	*p*	AUC	^1^Effect index	Cut off
QLQ-C30						
Global health	50.6 (42.1-59.2)	52.3 (42.0- 62.7)		0.5 (0.4-0.7)	0.1	
Functionality						
Physical	79.2 (71.2-87.3)	72.9 (64.3-81.6)		0.6 (0.4-0.7)	0.3	
Role	52.6 (38.1-67.0)	50.5 (36.9-64.1)		0.5 (0.4-0.7)	0.1	
Cognitive	84.0 (75.6-98.4)	70.8 (60.8-80.8)	0.040	0.7 (0.5-0.8)	0.5	< 83.33
Social	82.7 (71.7-93.7)	65.8 (53.3-78.2)	0.022	0.7 (0.5-0.8)	0.5	< 83.33
Emotional	68.6 (58.2-78.9)	75.8 (67.3-84.2)		0.6 (0.4-0.7)	0.3	
Symptoms						
Pain	21.8 (12.9-30.7)	37.5 (26.3-48.7)	0.014	0.7 (0.5-0.8)	0.6	> 37.50
Fatigue	46.2 (32.5-59.8)	47.6 (37.0-58.2)		0.6 (0.4-0.7)	0.1	
Loss of appetite	35.9 (21.2-50.5)	47.9 (33.6-62.2)		0.6 (0.5-0.8)	0.3	
Nausea	16.7 (7.0-26.4)	21.9 (11.4-32.4)		0.5 (0.4-0.7)	0.2	
Diarrhea	32.1 (17.1-47.0)	22.9 (12.6-33.2)		0.6 (0.4-0.7)	0.3	
Constipation	9.0 (2.9-15.1)	20.8 (10.4-31.3)		0.6 (0.4-0.7)	0.5	
Dyspnoe	16.7 (4.5-28.9)	15.6 (5.1-26.2)		0.5 (0.4-0.7)	0.0	
Sleep disturbance	37.2 (23.3-51.1)	39.6 (28.0-51.2)		0.5 (0.4-0.7)	0.1	
QLC-PAN26						
Pancreatic pain	20.8 (12.7-28.9)	33.9 (25.3-42.6)	0.025	0.7 (0.5-0.8)	0.5	> 31.25
Back pain	25.6 (11.2-40.1)	29.3 (16.5-42.1)		0.6 (0.4-0.7)	0.1	
Night pain	9.0 (1.8-16.2)	27.4 (17.4-37.4)	0.003	0.7 (0.6-0.8)	0.7	> 16.67
Digestive function	35.3 (21.5-49.0)	54.2 (39.6-68.7)		0.6 (0.5-0.8)	0.5	
Altered bowel habit	30.8 (16.8-44.7)	23.2 (13.4-33.1)		0.5 (0.4-0.7)	0.2	
Flatulence	25.6 (14.0-37.3)	34.5 (22.1-47.0)		0.5 (0.4-0.7)	0.3	
Hepatic symptoms	32.7 (16.6-48.8)	19.6 (9.2-30.1)		0.6 (0.4-0.7)	0.5	
Change of taste	23.1 (11.2-35.0)	41.7 (24.6-58.8)		0.6 (0.5-0.8)	0.5	
Dry mouth	29.5 (16.1-42.9)	48.8 (36.9-60.7)	0.027	0.7 (0.5-0.8)	0.5	> 33.33
Weakness	24.4 (13.9-34.8)	31.3 (19.9-42.7)		0.6 (0.4-0.7)	0.2	
Low activity	34.6 (21.2-48.1)	44.8 (32.0-57.6)		0.6 (0.4-0.7)	0.3	

^1^Cohen’s d effect index: 0.2: small effect, 0.5: medium effect, 0.8: large effect

In the univariate analysis, there was only an association between DSS and night pain in the QLQ- PAN26 questionnaire. After multivariate analysis by stepwise regression and backward elimination, an association between night pain and RFS also remained in the model. In patients with night pain and a score ≥ 66.66 the hazard ratio of dying was 2.0 (CI 0.64–6.21) times higher (*p* = 0.034). Adjusted analyses for co-variates (age, ASA, diabetes sex, tumor and lymph node stage) only showed association between outcome and lymph node stage.

Fifty-three patients completed adjuvant oncologic treatment, gemcitabine, or gemcitabine and capecitabine in patients with PDAC and FOLFOX in patients with intestinal derived adenocarcinoma.

**Fig. 2:**
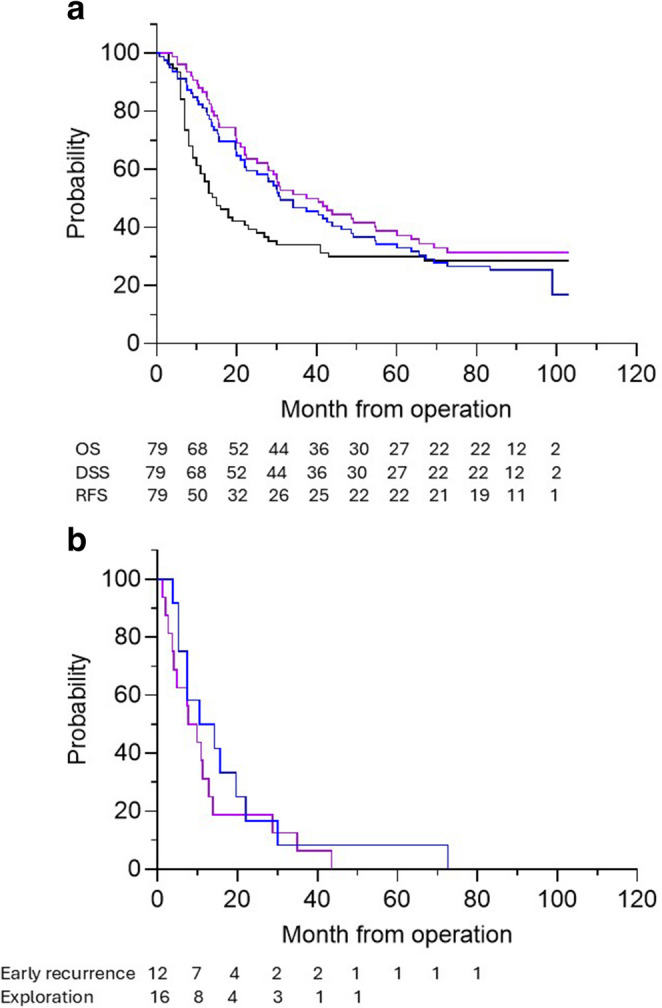
**A**. Overall survival (OS ── Black), disease specific survival (DSS ── Blue) and recurrence free survival (RFS ── Purple) of patients after pancreatic resection for pancreatic and periampullary adenocarcinoma **B**. Overall survival of patients after exploratory laparotomy (Early recurrence ── Blue) and recurrence (Exploratory laparotomy ── Purple) less than six months from pancreatic resection for adenocarcinoma

The median follow-up time of all 79 patients after pancreatic resection was 30.9 (0.7–103.1.7.1) months without drop-outs. The median OS, DSS, and RFS were 30.9 (0.7–103.1.7.1), 40.5 (1.9–103.1.9.1), and 15.0 (3.0–67.0) months, respectively (Fig. 2A). The recurrence rate of adenocarcinoma was 66% with a median time to recurrence of 9 (3.0–67.0) months. Patients with early recurrence and unresectable disease had a median survival of 12.4 (3.9–72.7) and 8.0 (1.0–43.0) months, respectively (*p* = 0.200)(Fig. 2B).

All patients with recurrence except two died during the observation period, median time to death was 22 (3.0–67.0) months. Two patients, who had liver metastases after Whipple’s operation for PDAC, have survived recurrence so far. The first patient developed liver metastases 10 months after the operation, but after four years and 48 series of FOLFIRINOX metastases had disappeared, and the patient was still alive at the end of the study. The second patient had a single metastasis that was treated with radiofrequency ablation, and the patient is without recurrence 70 months after this procedure.

**Fig. 3:**
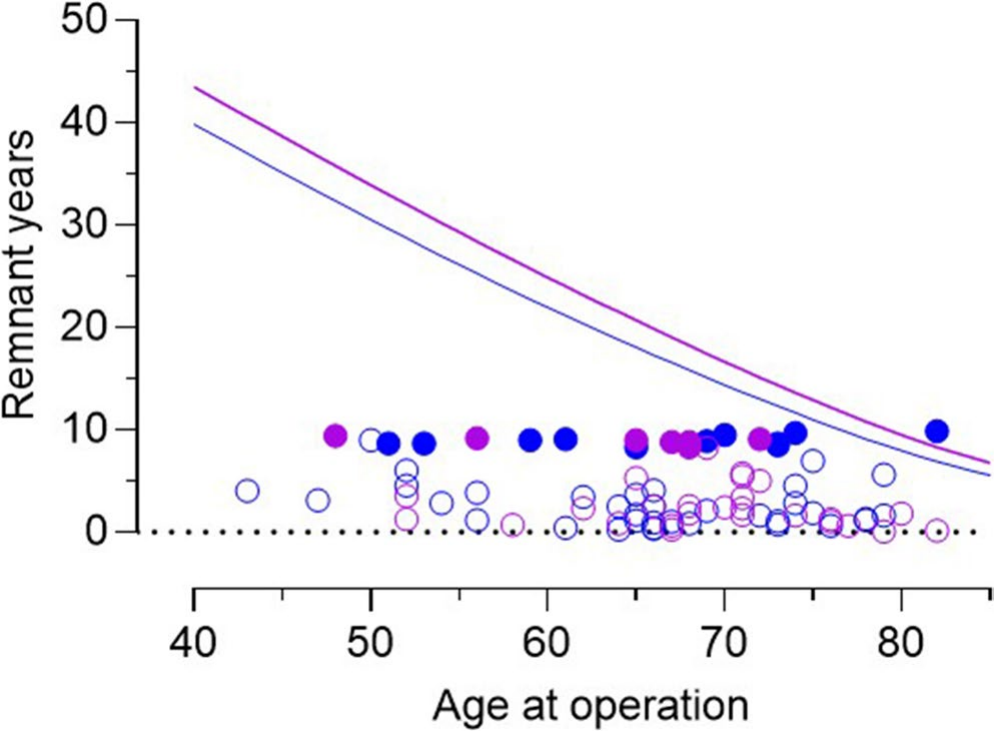
Lost remnant life of 79 men and women after pancreatic surgery for pancreatic and periampullary adenocarcinoma. Open and closed circles represent deceased patients and survivors. The solid lines are the expected remnant life of men (blue) and women (purple) between 40 and 85 years in a Danish standard population (www.dst.dk)

At the end of follow-up, two patients (11.7%) with benign disease and 62 (78.5%) patients with adenocarcinoma had died after radical surgery, 10 of them from conditions unrelated to the primary disease. The median lost remnant life of all patients after surgical resection for adenocarcinoma was 14.7 (2.9–32.9) months without significant gender difference (Fig. 3).

## Discussion

The preoperative QoL scores showed significant differences in several domains in both the QLQ-C30 and QLQ-PAN26 questionnaires between patients with benign pathology and adenocarcinoma. The deviations were present within the functionality as well as the symptom scores and were particularly prominent in patients who had exploratory laparotomy due to advanced disease.

Most QoL studies of patients who have undergone surgery for cancer are primarily concerned with the patients’ needs, either based on a postoperative assessment or based on a comparison of the preoperative and postoperative general condition. However, there are only limited studies on the association between preoperative QoL and OS or RSF, and only a few of them in patients with pancreatic cancer.

A French study of patients who underwent pancreatic surgery for PDAC and benign diseases found significant deviations in the QLQ-C30 scores between the two groups of patients [23]. The patients were evaluated every six months postoperatively for two years, and differences were found in domains such as physical function, role, social function, fatigue, pain, and loss of appetite, but there was no estimation of OS and RFS in relation to symptom score, nor did the study include patients who underwent only exploratory laparotomy. Although the set-up was different from our study, this is the only study apart from our that compared patients with either cancer or benign disease of the pancreas.

When we compared patients with advanced disease or early recurrence - arbitrarily defined as within six months - and patients without recurrence, we found significant differences in the cognitive and social function as well as pain in the QLQ-C30 score and in the domains pancreatic pain, night pain, and dry mouth in the QLQ-PAN26 questionnaire. The AUC of the ROC curve showed best agreement with pain and night pain equivalent to the Cohen’s d effect index. AUC values below 0.8 are of limited utility in biological science, in population science, however, values down to 0,7 are often considered acceptable, as high individual variability and overlapping risk distributions make perfect separation difficult. Small sample size, as in our study, may result in stepped ROC curves, which may yield low AUC. This implies uncertainty about the estimated cutoff values. Other methods based on simulated data and bootstrapping have been proposed [24, 25], but with the rather small sample size and the big variance in our data, this method may create more bias than valid results.

While there was a significant difference between our patients with benign disease and cancer, the associations between the preoperative scores and DSS and RFS were less clear. Again, only night pain was associated with DSS and RFS in the univariate analysis and was the only domain that remained after multivariate analysis with backward stepwise regression.

Our findings are somewhat inconsistent with other studies where a greater or lesser degree of relationship between several domains and OS was found. These studies, however, were mainly postoperative surveys to evaluate the postoperative needs without the preoperative QOL as baseline [26–29]. A postoperative decline in QoL for weeks or months is expected after pancreatic surgery, and it may be difficult to distinguish between postoperative fatigue and symptoms from early recurrence or undiagnosed disseminated disease present at the operation.

A Dutch study of 6.895 patients from an ongoing database including 12 cancer types except pancreatic cancer over a period of seven years found that the QLQ-C30 summary score was the strongest predictor of all-cause mortality compared with the global QoL score and the physical functioning score [30]. Analysis of the functionality scores and symptoms showed a variable association with all-cause mortality depending on the malignant disease and a difference in outcome relative to cancer site. Although the questionnaire was implemented up to six months from the diagnosis and completed up to two years later without preoperative data, it is noteworthy that different cancer types gave different results in the QoL scores.

The difference between cancer sites and the QoL scores has also been reported by other investigators. In studies of patients with lung cancer and meta-analyses of patients with gastrointestinal cancer, the pretreatment domains global, physical, social, role, emotional and pain were variably associated with OS [31–33].

In one of the few studies on preoperative QoL and OS in pancreatic cancer, cognitive function was the only domain associated with a shorter survival in both univariate and multivariate analysis [10]. Comparable results were found in a study of esophageal cancer [34], and in an all-round study of elderly onco-surgical patients [35] but not in a meta-analysis of patients with breast cancer [36].

The deviations in outcomes and the variation between different malignancies in relation to the QoL scores may indicate a basic discrepancy between subjective parameters in the questionnaires and objective parameters such as recurrence and survival. Various types of cancer may have different scores in the individual domains and ethnicity can have an impact on the results as well [37]. There may also be a difference in how patients with pancreatic cancer perceive their situation compared to patients with other malignancies. Although cancer has a significant impact on functionality, it is noteworthy that many studies primarily found deviations within role, cognitive function, emotional and social function in relation to OS rather than deviations within common cancer symptoms like fatigue, weakness, pain, and loss of appetite, but functionality may be of greater importance to patients during the completion of the questionnaire.

PDAC is especially known for its debilitating symptom burden which has a profound negative effect on QoL. However, there are also patients who do not have cancer related symptoms but nevertheless belong to the unfortunate group of 60% of patients with recurrence during the first postoperative year [38]. This unpredictable course may explain why the preoperative QoL scores and RFS and DSS had a weak correlation in our patients who had pancreatic resection, compared to patients with other malignant diseases, where a better association between QoL scores and outcome has been found. Moreover, time to recurrence among our patients varied from a few months to more than one year, which may also have an impact on the results. Only when we compared patients, who had early recurrence after pancreatic resection or who unexpectedly only had surgical exploration due to advanced disease, a significant deviation from patients with a better outcome was found.

If a summary assessment of the existing publications on preoperative QoL is made, there is generally no consistency between domains in the QLQ-C30 and PAN26 questionnaire and OS in patients with cancer. Some relations were even questionable and gave an impression of random correlation. Instead, there is a large individual variation that depends on the patients’ current condition at the time of the survey. In this context it should be emphasized that the way we and other researchers have tried to use the questionnaires in a prognostic setting does not correspond to what they were originally designed for. This means that domains which are important for the evaluation of patients’ post-operative well-being and needs may be less suitable for predicting cancer recurrence and survival in a pre- or postoperative setting.

The strength of our study was the prospective design of a homogenous group of patients with cancer or benign disease of the pancreas and periampullary area undergoing a standardized treatment and a long-time follow-up. Moreover, the completion rate of the questionnaires was high. The single setting ensured a standardized evaluation and follow-up, and the questionnaires and patients’ contact were managed by the same investigator.

A major limitation was the relatively small number of patients in the different groups, which may have had an impact on the statistical evaluation as some changes were disproportional regarding size and significance. This made the estimation of cut points of the QoL scores less reliable. Another limitation that applies to all qualitative studies is whether the patients have understood the questionnaires to provide valid answers.

## Conclusion

Our conclusion is that the EORTC QLQ-C30 and EORTC QLQ-PAN26 questionnaires found a significant difference between the QoL scores of patients operated on for benign disease and cancer, and especially patients with non-resectable disease. This also applied to patients with advanced disease or early recurrence versus patients without recurrence. The questionnaires might be considered for preoperative evaluation of early postoperative recurrence and may be supplemental to clinical assessments, but an adjustment of the questionnaires for this purpose seems warranted.

## Data Availability

No datasets were generated or analysed during the current study.
